# Author Correction: The Aurora B specificity switch is required to protect from non-disjunction at the metaphase/anaphase transition

**DOI:** 10.1038/s41467-020-16468-2

**Published:** 2020-06-03

**Authors:** Joanna R. Kelly, Silvia Martini, Nicola Brownlow, Dhira Joshi, Stefania Federico, Shirin Jamshidi, Svend Kjaer, Nicola Lockwood, Khondaker Miraz Rahman, Franca Fraternali, Peter J. Parker, Tanya N. Soliman

**Affiliations:** 1 Protein Phosphorylation Laboratory, Francis Crick Institute, 1 Midland Rd, London, NW1 1AT UK; 2 Peptide Chemistry Platform, Francis Crick Institute, 1 Midland Rd, London, NW1 1AT UK; 3 School of Cancer and Pharmaceutical Sciences, King’s College London, London, UK; 4 Structural Biology Platform, Francis Crick Institute, 1 Midland Rd, London, NW1 1AT UK; 5 Randall Centre for Cell and Molecular Biophysics, King’s College London, London, UK; 6 Present Address: Cancer Research UK, Manchester Institute, Alderley Park, SK10 4TG UK; 7 Present Address: Instituto de Neurociencias, Av. Santiago Ramón y Cajal s/n, 03550 San Juan de Alicante, Spain; 8 Present Address: Barts Cancer Institute, Queen Mary University London, Charterhouse Square, London, EC1M 6BE UK

**Keywords:** Kinases, Mitosis, Cell signalling, Phosphorylation

Correction to: *Nature Communications* 10.1038/s41467-020-15163-6, published online 13 March 2020.

The original version of this Article contained an error in the spelling of the author Khondaker Miraz Rahman, which was incorrectly given as Khondaker Miraz Rahmen. This has now been corrected in both the PDF and HTML versions of the Article.

