# Supplementary material for: The Aurora B specificity switch is required to protect from non-disjunction at the metaphase/anaphase transition
Source: Nat Commun. 2020 Mar 13;11:1396. doi: 10.1038/s41467-020-15163-6 (PMC7070073; doi:10.1038/s41467-020-15163-6)
Supplement: Supplementary file 3 — Reporting Summary [file 41467_2020_15163_MOESM3_ESM.pdf]

nature research

Corresponding author(s): Peter Parker/Tanya Soliman

Last updated by author(s): Feb 13, 2020

Reporting Summary

Nature Research wishes to improve the reproducibility of the work that we publish. This form provides structure for consistency and transparency in reporting. For further information on Nature Research policies, see Authors & Referees and the Editorial Policy Checklist .

Statistics

For all statistical analyses, confirm that the following items are present in the figure legend, table legend, main text, or Methods section.

n/a	Confirmed	
☐	☒	The exact sample size (*n*) for each experimental group/condition, given as a discrete number and unit of measurement
☐	☒	A statement on whether measurements were taken from distinct samples or whether the same sample was measured repeatedly
☐	☒	The statistical test(s) used AND whether they are one- or two-sided<br>*Only common tests should be described solely by name; describe more complex techniques in the Methods section.*
☐	☒	A description of all covariates tested
☐	☒	A description of any assumptions or corrections, such as tests of normality and adjustment for multiple comparisons
☐	☒	A full description of the statistical parameters including central tendency (e.g. means) or other basic estimates (e.g. regression coefficient) AND variation (e.g. standard deviation) or associated estimates of uncertainty (e.g. confidence intervals)
☐	☒	For null hypothesis testing, the test statistic (e.g. *F*, *t*, *r*) with confidence intervals, effect sizes, degrees of freedom and *P* value noted<br>*Give P values as exact values whenever suitable.*
☒	☐	For Bayesian analysis, information on the choice of priors and Markov chain Monte Carlo settings
☒	☐	For hierarchical and complex designs, identification of the appropriate level for tests and full reporting of outcomes
☒	☐	Estimates of effect sizes (e.g. Cohen's *d*, Pearson's *r*), indicating how they were calculated

Our web collection on statistics for biologists contains articles on many of the points above.

Software and code

Policy information about availability of computer code

Data collection	Image-J 1.50i analysis software<br>MetaMorph (Version 6.3)<br>Zen (Version 2.3 SP1)<br>ImageQuant Las 4000 (Version 1.1)<br>VisionWorks analysis software - Analytik Jena (version 4.12.16131.914)<br>custom-built script in MATLAB (MATLAB R2018a)<br>SwissModel (web server)<br>AMBER 16 (Version 16 of the Amber Molecular Dynamics Package)<br>HPEPDOCK web server (webserver, version 08/2019)<br>Pep-SiteFinder (webserver)
Data analysis	Prism software - Graphpad (version 7.0c)

For manuscripts utilizing custom algorithms or software that are central to the research but not yet described in published literature, software must be made available to editors/reviewers. We strongly encourage code deposition in a community repository (e.g. GitHub). See the Nature Research guidelines for submitting code & software for further information.

Data

Policy information about availability of data

All manuscripts must include a data availability statement. This statement should provide the following information, where applicable:

- Accession codes, unique identifiers, or web links for publicly available datasets
- A list of figures that have associated raw data
- A description of any restrictions on data availability

The data that support the findings of this study are available from the corresponding author upon reasonable request.

Field-specific reporting

Please select the one below that is the best fit for your research. If you are not sure, read the appropriate sections before making your selection.

[x] Life sciences [ ] Behavioural & social sciences [ ] Ecological, evolutionary & environmental sciences

For a reference copy of the document with all sections, see nature.com/documents/nr-reporting-summary-flat.pdf

Life sciences study design

All studies must disclose on these points even when the disclosure is negative.

Sample size	Sample sizes used in the study were designed based on the sample size required to produce robust statistics from the relevant statistical methods used in each experiment. Sample sizes were chosen for the differing experimental approaches based on the technical difficulty and throughput of the individual assays.
Data exclusions	No data was excluded from the manuscript.
Replication	All reported data was found to be reproducible in this study.<br>For the majority of data presented a minimum of 3 independent experimental repeats were performed using to ensure reproducibility of the results.<br>The exception to this is the peptide array. The array was used as a screening tool, the results of which were validated in subsequent experiments.
Randomization	Randomization was not applicable to the study as all experiments were performed using immortalized cell lines. The assays used in this study are not subject to the systematic variation which demands randomisation.
Blinding	Blinding was used for analysis of catenation spread experiments, in which scoring was performed manually. Individual images from the data sets were assigned random numbers by a colleague who was otherwise uninvolved in the experiment and re-sorted into the appropriate experimental groups post analysis.<br>For experiments where data scoring/analysis was performed using by automated means using pre-defined software blinding was not deemed necessary.

Reporting for specific materials, systems and methods

We require information from authors about some types of materials, experimental systems and methods used in many studies. Here, indicate whether each material, system or method listed is relevant to your study. If you are not sure if a list item applies to your research, read the appropriate section before selecting a response.

Materials & experimental systems

n/a	Involved in the study	
[ ]	[x]	Antibodies
[ ]	[x]	Eukaryotic cell lines
[x]	[ ]	Palaeontology
[x]	[ ]	Animals and other organisms
[x]	[ ]	Human research participants
[x]	[ ]	Clinical data

Methods

n/a	Involved in the study	
[x]	[ ]	ChIP-seq
[x]	[ ]	Flow cytometry
[x]	[ ]	MRI-based neuroimaging

Antibodies

Antibodies used	mouse anti-TopoIIa (clone KiS1) - MAB4197, Millipore<br>rabbit anti-TopoIIa phosphoSer29 - polyclonal, made in house<br>rabbit anti-TopoIIa phosphoThr1460 - polyclonal, made in-house

mouse anti-alpha tubulin - clone DM1A, T9026, Sigma
rabbit anti-PICH - H00054821-D01, Abnova
mouse anti-Lap2B, 611000, BD
rabbit anti-Bub1 - ab9000, Abcam
rabbit anti-BubR1 - ab70544 , Abcam
rabbit anti-MAD2 - A300-300A, Bethyl Antibodies
human anti-centromere (CREST) - 15-234, Antibodies Inc
mouse anti-Aurora B - AIM-1, 611082, BD
rabbit anti-Aurora B phosphoThr232, TA319253, Origene
rabbit anti-Aurora B phosphoSer227, Polyclonal, made in-house
rabbit anti-Histone H3 - 9715, Cell Signaling
rabbit anti-Histone H3 phosphoSer10 - 9701, Cell Signaling
rabbit anti PKCe - sc214, Santa Cruz
rabbit anti-PKCe phosphoSer729 - ab63387, Abcam
rabbit anti-PKCe phosphoThr566 - polyclonal, made in-house
rabbit anti-PKCe phosphoThr710 - polyclonal, made in-house
mouse anti-GFP (clone 3E1)- monoclonal, made in-house
rabbit anti-GFP - ab290, Abcam
rabbit anti-Caspase 3 - AB1899, Millipore
mouse anti-Caspase 7 - 551238, BC Biosciences
mouse anti-GAPDH (clone 6C5) - MAB374, Millipore
anti-cleaved PARP Asp241 - 9541, Cell Signaling
anti-mouse HRP conjugated secondary antibody - NA931, GE Lifesciences
anti-rabbit HRP conjugated secondary antibody - NA934, GE Lifesciences
Goat anti-mouse AlexaFluor 488 Conjugated secondary antibody - A11001, Life technologies
Goat anti-rabbit AlexaFluor 488 Conjugated secondary antibody - A11008, Life technologies
Goat anti-mouse AlexaFluor 555 Conjugated secondary antibody - A21422, Life technologies
Goat anti-rabbit AlexaFluor 555 Conjugated secondary antibody - A21428, Life technologies
Goat anti-mouse AlexaFluor 647 Conjugated secondary antibody - A32728, Life technologies
Goat anti-rabbit AlexaFluor 647 Conjugated secondary antibody - A21244, Life technologies
Goat anti-human AlexaFluor 647 Conjugated secondary antibody - A21445, Life technologies
Rabbit IgG - SC2027, Santa Cruz
Mouse IgG - SC2025, Santa Cruz

Validation

mouse anti-TopoIIa - MAB4197, Millipore (https://www.merckmillipore.com/GB/en/product/Anti-Topoisomerase-II-Antibody-clone-KiS1,MM_NF-MAB4197)

rabbit anti-TopoIIa phosphoSer29 - polyclonal, made in house, see manuscript Figure 4A,B,E-G, 5C, Supplementary Figure 5A-D

rabbit anti-TopoIIa phosphoThr1460 - polyclonal, made in-house, see manuscript Figure 4E, Supplementary Figure 5B, E-F

mouse anti-alpha tubulin - clone DM1A, T9026, Sigma (https://www.sigmaaldrich.com/catalog/product/sigma/t9026?lang=en&region=GB)

rabbit anti-PICH - H00054821-D01, Abnova (http://www.abnova.com/products/products_detail.asp?catalog_id=H00054821-D01)

mouse anti-Lap2B, 611000, BD (https://www.bdbiosciences.com/us/reagents/research/antibodies-buffers/cell-biology-reagents/cell-biology-antibodies/purified-mouse-anti-lap2-27lap2/p/611000)

rabbit anti-Bub1 - ab9000, Abcam (https://www.abcam.com/bub1-antibody-ab9000.html)

rabbit anti-MAD2 - A300-300A, Bethyl Antibodies (https://www.bethyl.com/product/A300-300A/MAD2+Antibody)

rabbit anti-BubR1 - ab70544 , Abcam (https://www.abcam.com/bubr1-antibody-ab70544.html)

human anti-centromere (CREST) - 15-234, Antibodies Inc (Serum obtained from an autoimmune patient was tested at a series of dilutions by immunocytochemistry on ethanol-fixed Hep2 cells that were in log-phase growth. The staining pattern obtained was consistent with the pattern expected for anti-centromere staining.)

mouse anti-Aurora B - AIM-1, 611082, BD (https://www.bdbiosciences.com/us/reagents/research/antibodies-buffers/cell-biology-reagents/cell-biology-antibodies/purified-mouse-anti--aim-1-6aim-1/p/611082)

rabbit anti-Aurora B phosphoThr232, TA319253, Origene (https://www.origene.com/catalog/antibodies/primary-antibodies/ta319253/aurora-b-aurkb-rabbit-polyclonal-antibody)

rabbit anti-Aurora B phosphoSer227, Polyclonal, made in-house, (See Pike T et al., Nature Communications, 2016)

rabbitt anti-Histone H3 - 9715, Cell Signaling (https://www.cellsignal.co.uk/products/primary-antibodies/histone-h3-antibody/9715)

rabbit anti-Histone H3 phosphoSer10 - 9701, Cell Signaling (https://www.cellsignal.co.uk/products/primary-antibodies/phospho-histone-h3-ser10-antibody/9701?_=1581465567157&Ntt=9701&tahead=true)

rabbit anti PKCe - sc214, Santa Cruz (https://www.scbt.com/p/pkc-epsilon-antibody-c-15)

rabbit anti-PKCe phosphoSer729 - ab63387, Abcam (https://www.abcam.com/pkc-epsilon-phospho-s729-antibody-ab63387.html)

rabbit anti-PKCe phosphoThr566 - polyclonal, made in-house (see Cameron A et al., Nature Structural and Molecular Biology, 2009)

rabbit anti-PKCe phosphoThr710 - polyclonal, made in-house (see Cameron A et al., Nature Structural and Molecular Biology, 2009)

mouse anti-GFP (clone3E1)- monoclonal, made in-house

rabbit anti-GFP - ab290, Abcam (https://www.abcam.com/gfp-antibody-chip-grade-ab290.html)

rabbit anti-Caspase 3 - AB1899, Millipore (https://www.merckmillipore.com/GB/en/product/Anti-Caspase-3-Antibody-large-subunit-proform,MM_NF-AB1899?ReferrerURL=https%3A%2F%2Fwww.google.com%2F&bd=1)

mouse anti-Caspase 7 - 551238, BD Biosciences (https://www.bdbiosciences.com/us/applications/research/apoptosis/purified-antibodies/purified-mouse-anti-caspase-7-10-1-62/p/551238)

mouse anti-GAPDH - MAB374, Millipore (https://www.merckmillipore.com/GB/en/product/Anti-Glyceraldehyde-3-Phosphate-Dehydrogenase-Antibody-clone-6C5,MM_NF-MAB374)

anti-cleaved PARP Asp241 - 9541, Cell Signaling (https://www.cellsignal.co.uk/products/primary-antibodies/cleaved-parp-asp214-antibody-human-specific/9541)

Eukaryotic cell lines

Policy information about cell lines

Cell line source(s)	DLD1 (ATCC)<br>DLD1-FRT-TRex (Prof. Stephen Taylor)<br>HEK293-FRT-TRex (Invitrogen)<br>HeLa (ATCC)<br>U20S (ATCC)<br>hTERT-RPE (ATCC)<br>A549 (ATCC)
Authentication	All cell lines were authenticated by the Francis Crick Institute by STR profiling
Mycoplasma contamination	Cell lines were routinely tested for mycoplasma. No contamination found.
Commonly misidentified lines<br>(See ICLAC register)	No commonly misidentified cell lines were used in this study.
