# Supplementary material for: The Aurora B specificity switch is required to protect from non-disjunction at the metaphase/anaphase transition
Source: Nat Commun. 2020 Mar 13;11:1396. doi: 10.1038/s41467-020-15163-6 (PMC7070073; doi:10.1038/s41467-020-15163-6)

SUPPLEMENTARY INFORMATION

THE AURORA B SPECIFICITY SWITCH IS REQUIRED TO PROTECT FROM NON-DISJUNCTION AT THE METAPHASE/ANAPHASE TRANSITION

KELLY JR, *et al.*

Supplementary Methods
Supplementary Figures 1-9

Supplementary Methods

Supplementary Table 1. Primers used for Cloning

Template	Vector	Primer sequence
T2A-GFP	pcDNA5	FW 5'AGCACAGTGGCGGCCGCATGGAAGTGTCACCATTGC3'<br>RV 5'CAGCGGGTTTAAACGGGCCCTTACTTGTACAGCTCGTCC3'

Supplementary Table 2. Primers used for site directed mutagenesis

Template	Mutation	Primer sequence
PKCε	D383N	FW 5'CTACAAGACGATGATGTGAACTGCACAATGACAGAGA3'<br>RV 5'TCTCTGTCATTGTGCAGTTCACATCATCGTCTTGTAG3'
PKCε	D451N	FW 5' TCGTCGGCCACCAATGGCCAGCTGG3'<br>RV 5'CCAGCTGGCCATTGGTGGCCGACGA3'
PKCε D383/451N	TEV insertion	FW 5' GGCCACCGATGAGAACCTGTACTTCCAGGGCCAGCTGG3'<br>RV 5' CCAGCTGGCCCTGGAAGTACAGGTTCTCATCGGTGGCC3'
T2A-GFP	S29A	FW 5'GCTAAGAAAAGACTGGCTGTTGAAAGAATCT3'<br>RV 5' AGATTCTTTCAACAGCCAGTCTTTTCTTAGC3'
T2A-GFP	T1460A	FW 5'CCTGATCCTGCCAAAGCCAAGAATCGCCGCA3'<br>RV'5'TGCGGCGATTCTTGGCTTTGGCAGGATCAGG3'

Supplementary Table 3. Primers for sequencing

Template	Primer sequence	Template	Primer sequence
CMV FWD	5'CGCAAATGGGCGGTAGGCGTG3'	TOP2A F7	5'CCAACTTTGATGTGCGTGAA3'
GFP FWD	5'CATGGTCCTGCTGGAGTTCGTG3'	TOP2A F8	5'TGGTGCTTTTTGACCACGTA3'
PKCε seq F1	5'TGGAGCCAGAAGGAAAAGTG3'	TOP2A F9	5'TGCCCCTTTGGTATTTAACC3'
PKCε seq F2	5'GCAAGCCCCGGAGAGAAC3'	TOP2A F10	5'GAAAAGAGAACCAGGTACAAAGACA3'
PKCε seq F3	5'CTCATGTTTCTCCACCAGCA3'	TOP2A F11	5'CATTTCCCAGATGAAACTGAAA3'
PKCε seq R1	5'GCTCCCTGAACACTCGTTCT3'	TOP2A F12	5'TTTTAAAATGTGAGGCGATT3'
PKCε seq R2	5'CTTTGCCCAACACCTTGATG3'	TOP2A R1	5'TTGTTGTCCGCAGCATTAAC3'
PKCε seq R3	5'5'GGGGCTTGATTTTCTTCTGC3'	TOP2A R2	5'GGCTTTGCATTTTAAACTTAGACA3'
TOP2A F1	5'ACGATGAAGATGTTGGCATT3'	TOP2A R3	5'TCCACATGTGATTTTTCACC3'
TOP2A F2	5'GGAAGAGCTGGTGAGATGGA3'	TOP2A R4	5'GAAGCTTCTCGAACATTGAGT3'
TOP2A F3	5'TGTGACTAAACTTGTTGATGTTGTG3'	TOP2A R5	5'TCTGCAAAGTATTCTTTAGCTTCC3'
TOP2A F4	5'AAACTTTGGCTGTTTCAGGC3'	TOP2A R6	5'TCACCATGATGATAAGAAGACATTT3'
TOP2A F5	5'TGGAAGAGTTCTACTCCAAATCA3'	TOP2A R7	5'TTCTTGTAACTTGGAAGCATTGG3'
TOP2A F6	5'TGCTTCAAACGGAATGACAA3'		

Supplementary Figure 1

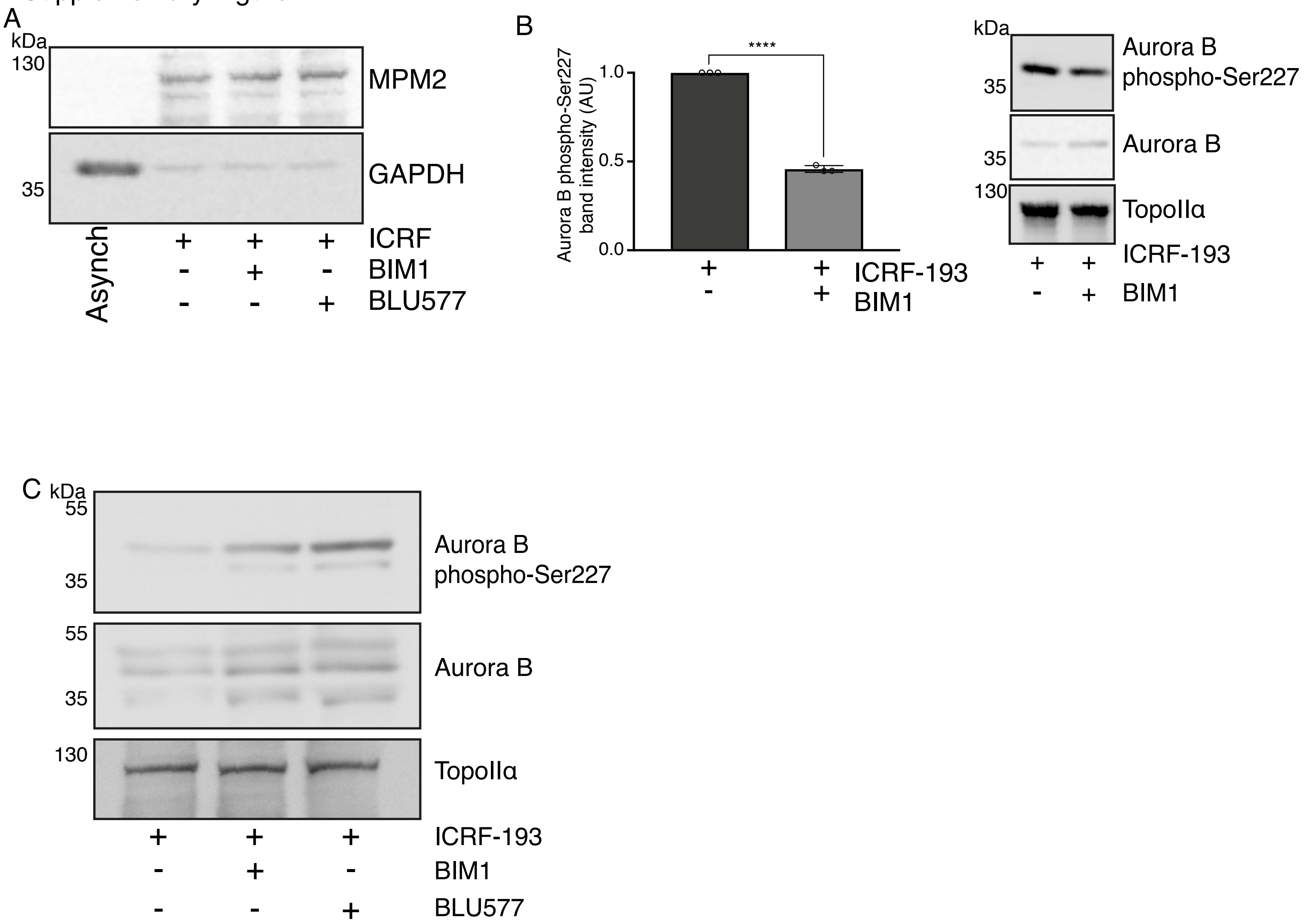

Supplementary Figure 1.

(A) Immunoblot of DLD1 cells asynchronous and enriched in mitosis by double thymidine block and RO-3306 + ICRF193 (1μM) arrest. After release, cells were treated with ±BLU577(500nM) or ± BIM1 (500nM). Triton-X100 soluble supernatant fractions were probed for MPM2 and GAPDH to demonstrate the mitotic enrichment of these samples. Immunoblot is representative of 3 independent experiments. (B) Immunoblot of DLD1 cells enriched in mitosis, treated with ICRF193 (1μM) ± BIM1 (500nM). Triton-X100 insoluble fractions were probed for Aurora B phospho-S227, total Aurora B and TopoIIα. Western blot is representative of 3 independent experiments, quantitative analysis of the immunoblots is demonstrated in the graph. (C) Immunoblot of asynchronous, DLD1 cells in log-phase growth. Cells were treated for 16 hours with ICRF193 (1μM) and ±BLU577 (500nM) or ± BIM1 (500nM) for 1 hour. Triton-X100 insoluble fractions were probed for Aurora B phospho-S227, total Aurora B and TopoIIα. Immunoblot is representative of 3 independent experiments.

Supplementary Figure 2

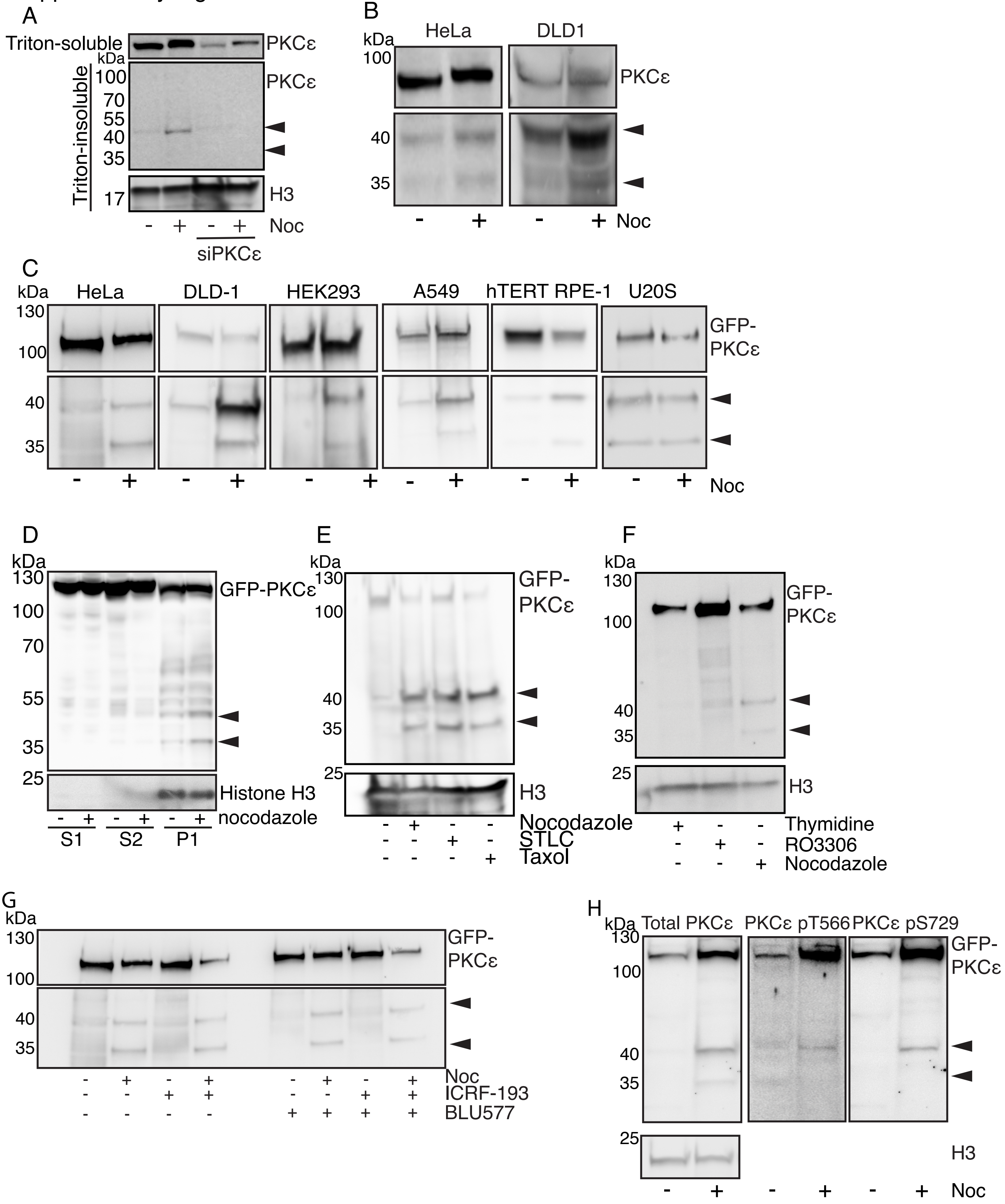

Supplementary Figure 2.

(A) DLD1 cells treated with ± 500nM nocodazole following 24h PKCε knock-down with siRNA were fractionated and lysates from Triton-soluble and Triton-insoluble fractions were probed for PKCε and Histone H3. Western blot is representative of 3 independent experiments. (B) Endogenous PKCε is cleaved in the Triton-insoluble fraction of nocodazole arrested HeLa and DLD1 cells. Western blot of PKCε is representative of 3 independent experiments. (C) GFP-PKCε is cleaved in the Triton-insoluble fraction of nocodazole arrested cells. Western blot of PKCε is representative of 3 independent experiments. (D) DLD1 cells were fractionated by high-speed centrifugation into the cytosolic (S1), nuclear (S2) and chromatin enriched (P1) compartments before immunoblotting for PKCε and Histone H3, representative of 3 independent experiments. (E) GFP-PKCε is cleaved in the Triton-insoluble fraction of DLD1 cells expressing GFP-PKCε arrested in mitosis with various inhibitors. Nocodazole (500nM), S-Trityl-L-Cysteine (1μM), Taxol (500nM). Western blot of PKCε and Histone H3 is representative of 3 independent experiments. (F) Immunoblot of Triton-insoluble fraction of DLD1 cells expressing GFP-PKCε arrested at G1/S phase with thymidine (2.5mM), G2 phase with RO-3306 (9μM) or prometaphase with nocodazole (500nM) and probed with PKCε and H3, representative of 3 independent experiments. (G) Immunoblot of Triton-insoluble fraction. DLD1 cells expressing GFP-PKCε were treated ± nocodazole (500nM) ± ICRF193 (1μM) ± Blu577 (500nM) and probed for PKCε, representative of 3 independent experiments. (H) Immunoblot of Triton-insoluble fraction of DLD1 cells expressing GFP-PKCε ± nocodazole (500nM) and probed with PKCε, PKCε pS729 or pT566 to observe phosphorylation of PKCε priming sites, representative of 3 independent experiments.

Supplementary Figure 3

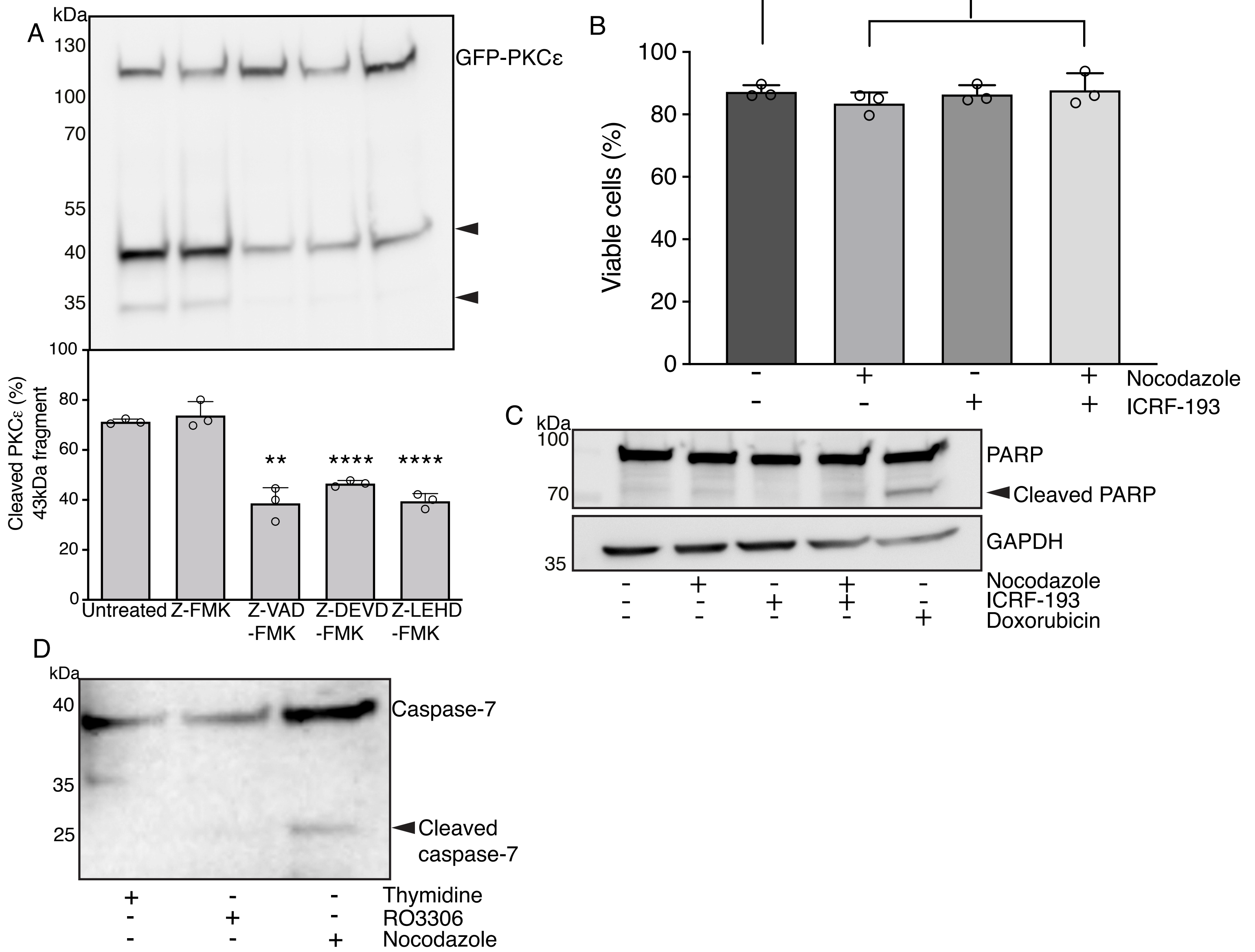

Supplementary Figure 3.
(A) Immunoblot of Triton-insoluble fraction treated with 10μM Caspase inhibitors (Z-VAD-FMK, Z-DEVD-FMK, Z-LEHD-FMK) and control peptide (Z-FMK), probed for PKCε. Graph denotes the mean ±SD of 3 independent experiments where the proportion of cleaved 43kDa fragment of PKCε present in immunoblots was determined by densitometry (One-way ANOVA, **P=0.0011 **** P=≤ 0.001, Untreated 71.33%±1.04, Z-FMK 73.87%±5.48, Z-VAD-FMK 38.67%±6.7, Z-DEVD-FMK 46.6%±1.20, Z-LEHD-FMK39.57%±2.94). (B) Cell viability was assessed using trypan blue dye exclusion after 24h treatment with nocodazole (500nM) ± ICRF193 (1μM). Graph represents the mean ±SD of three independent experiments. (C) Immunoblot of DLD1 cell lysates treated with nocodazole (500nM), ICRF193 (1μM) or doxorubicin (500nM) and probed for PARP and GAPDH, representative of 3 independent experiments. (D) Immunoblot of Thymidine (2.5mM), RO-3306 (9μM) and Nocodazole (500nM) arrested DLD1 cells probed with an antibody recognising both the pro- and active (cleaved) forms of Caspase 7, representative of 3 independent experiments.

Supplementary Figure 4

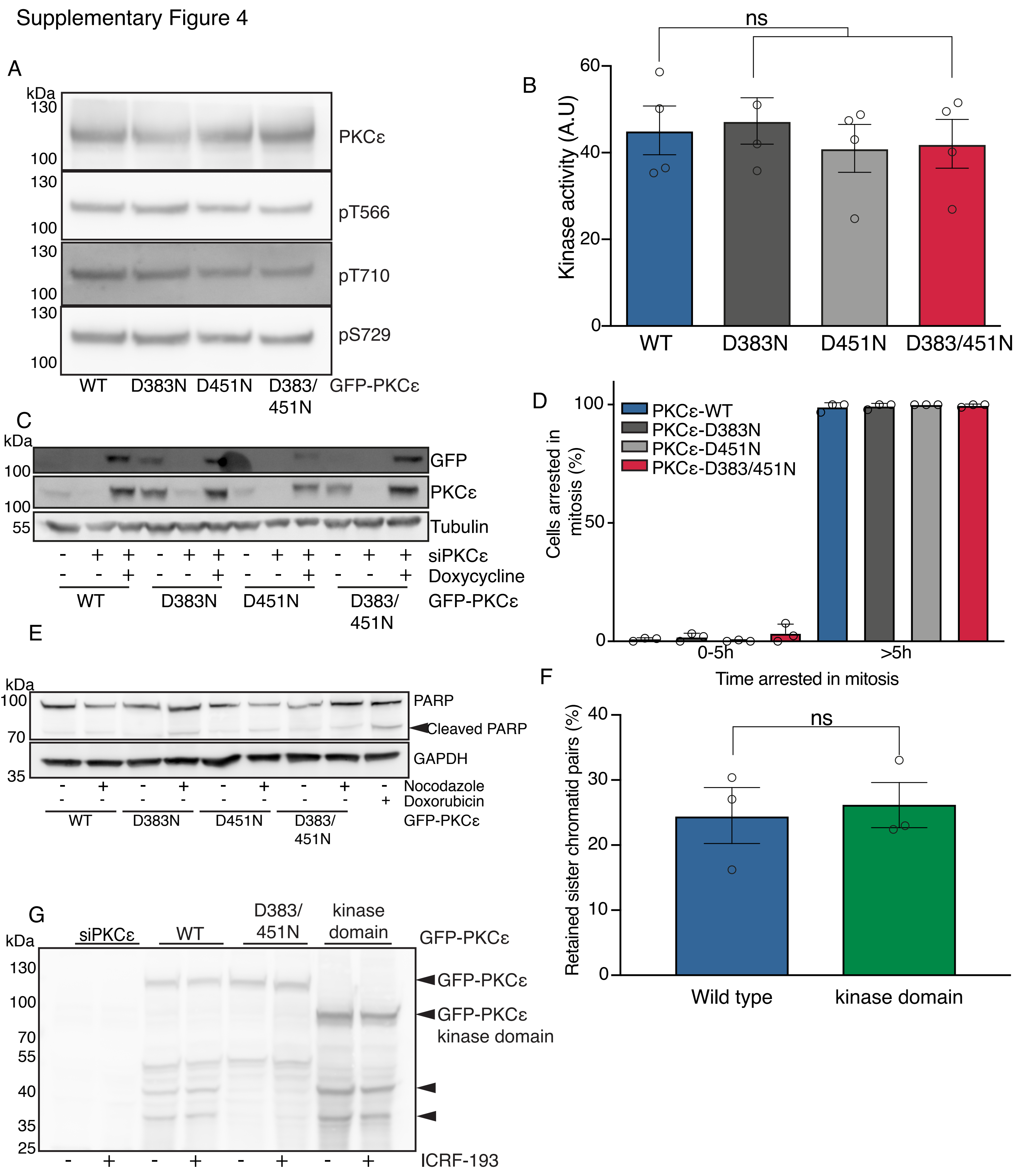

Supplementary Figure 4.

(A) Immunoblot of GFP-PKCε cleavage site mutants probed with PKCε, PKCε pS729, pT710 or pT566 to observe phosphorylation of priming sites, representative of 3 independent experiments. (B) Kinase activity of immunoprecipitated GFP-PKCε mutants assayed using ATP-Glo™. Graph represents the mean ±SD of three independent experiments (one-way ANOVA; WT 45.15±11.22, D383N 47.32±10.72, D451N 41±11.08, D383/451N 42.04±11.22). (C) Immunoblot (representative of 3 independent experiments) of DLD-1 FRT-TRex GFP-PKCε cell lines demonstrating siRNA depletion of endogenous PKCε protein and GFP expression only with doxycycline treatment for 24h. (D) SAC test of GFP-PKCε mutant expressing DLD1 cells. Cells were treated with 500nM nocodazole and imaged using live cell time lapse microscopy for 24 hours and scored for their time arrested in mitosis. The mean ±SD of 3 experiments where >50 cells were analysed per condition, per experiment are represented by the graph. (E) Immunoblot from lysates of DLD1 cells induced to express GFP-PKCε treated ± nocodazole (500nM) or doxorubicin (500nM) and probed for PARP and GAPDH, representative of 3 independent experiments.(F) Catenation spread assay analysis. Cells were scored for the per cent joined (catenated) chromatids as per representative images. The mean of three experiments ±SD where 15 cells per experiment were analysed is represented by the graph. (two-tailed Students' t-test; WT 24.55%±4.28, kinase domain 26.15%±3.45) (G) Immunoblot (representative of 3 independent experiments) of Triton-insoluble fraction of nocodazole arrested DLD1 cells expressing GFP-PKCε wild type, D383/451N or kinase domain ± ICRF193 (1μM).

Supplementary Figure 5

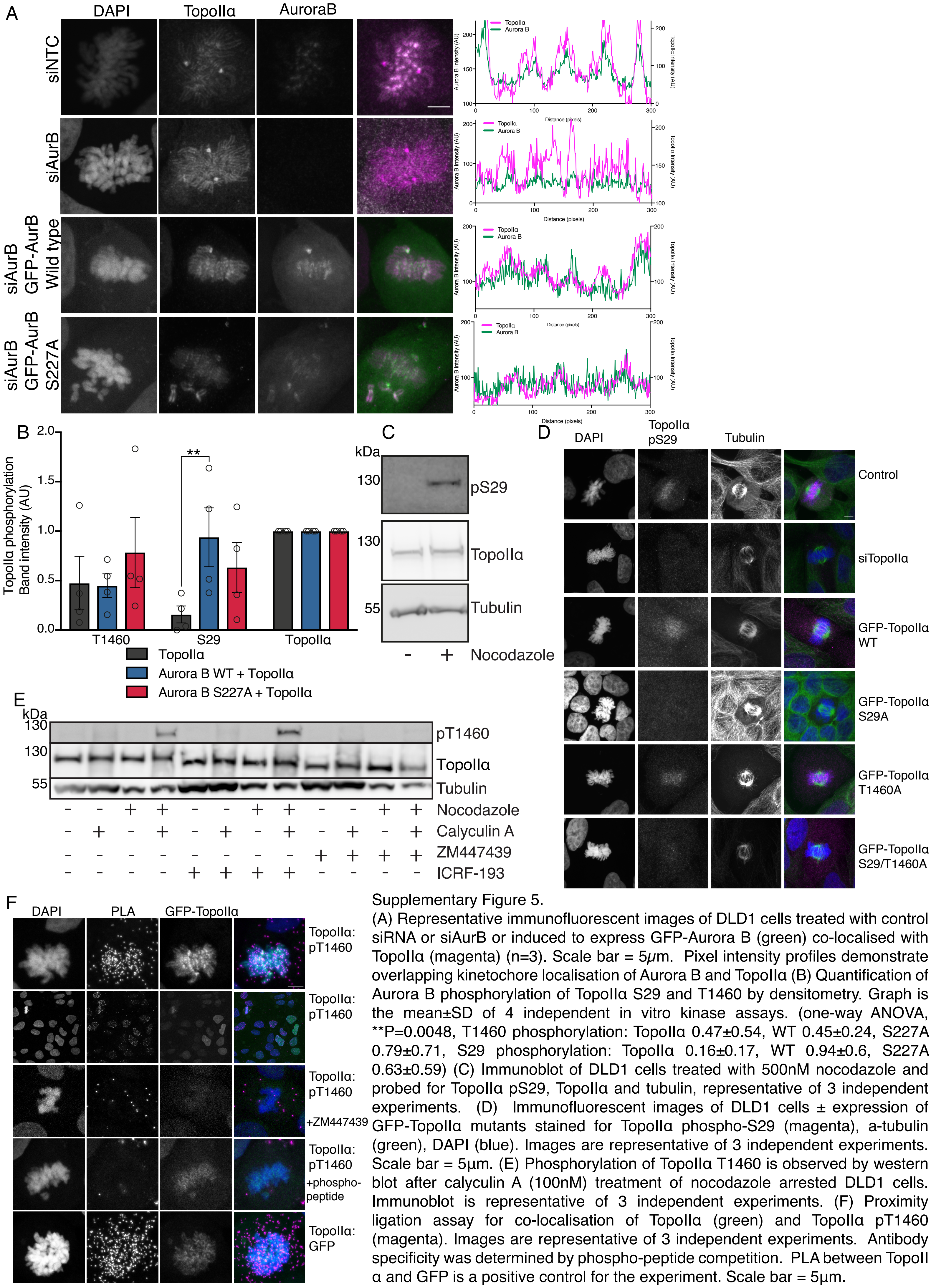

Supplementary Figure 5.
(A) Representative immunofluorescent images of DLD1 cells treated with control siRNA or siAurB or induced to express GFP-Aurora B (green) co-localised with TopoIIα (magenta) (n=3). Scale bar = 5μm. Pixel intensity profiles demonstrate overlapping kinetochore localisation of Aurora B and TopoIIα (B) Quantification of Aurora B phosphorylation of TopoIIα S29 and T1460 by densitometry. Graph is the mean±SD of 4 independent in vitro kinase assays. (one-way ANOVA, **P=0.0048, T1460 phosphorylation: TopoIIα 0.47±0.54, WT 0.45±0.24, S227A 0.79±0.71, S29 phosphorylation: TopoIIα 0.16±0.17, WT 0.94±0.6, S227A 0.63±0.59) (C) Immunoblot of DLD1 cells treated with 500nM nocodazole and probed for TopoIIα pS29, TopoIIα and tubulin, representative of 3 independent experiments. (D) Immunofluorescent images of DLD1 cells ± expression of GFP-TopoIIα mutants stained for TopoIIα phospho-S29 (magenta), a-tubulin (green), DAPI (blue). Images are representative of 3 independent experiments. Scale bar = 5μm. (E) Phosphorylation of TopoIIα T1460 is observed by western blot after calyculin A (100nM) treatment of nocodazole arrested DLD1 cells. Immunoblot is representative of 3 independent experiments. (F) Proximity ligation assay for co-localisation of TopoIIα (green) and TopoIIα pT1460 (magenta). Images are representative of 3 independent experiments. Antibody specificity was determined by phospho-peptide competition. PLA between TopoII α and GFP is a positive control for the experiment. Scale bar = 5μm.

Supplementary Figure 6

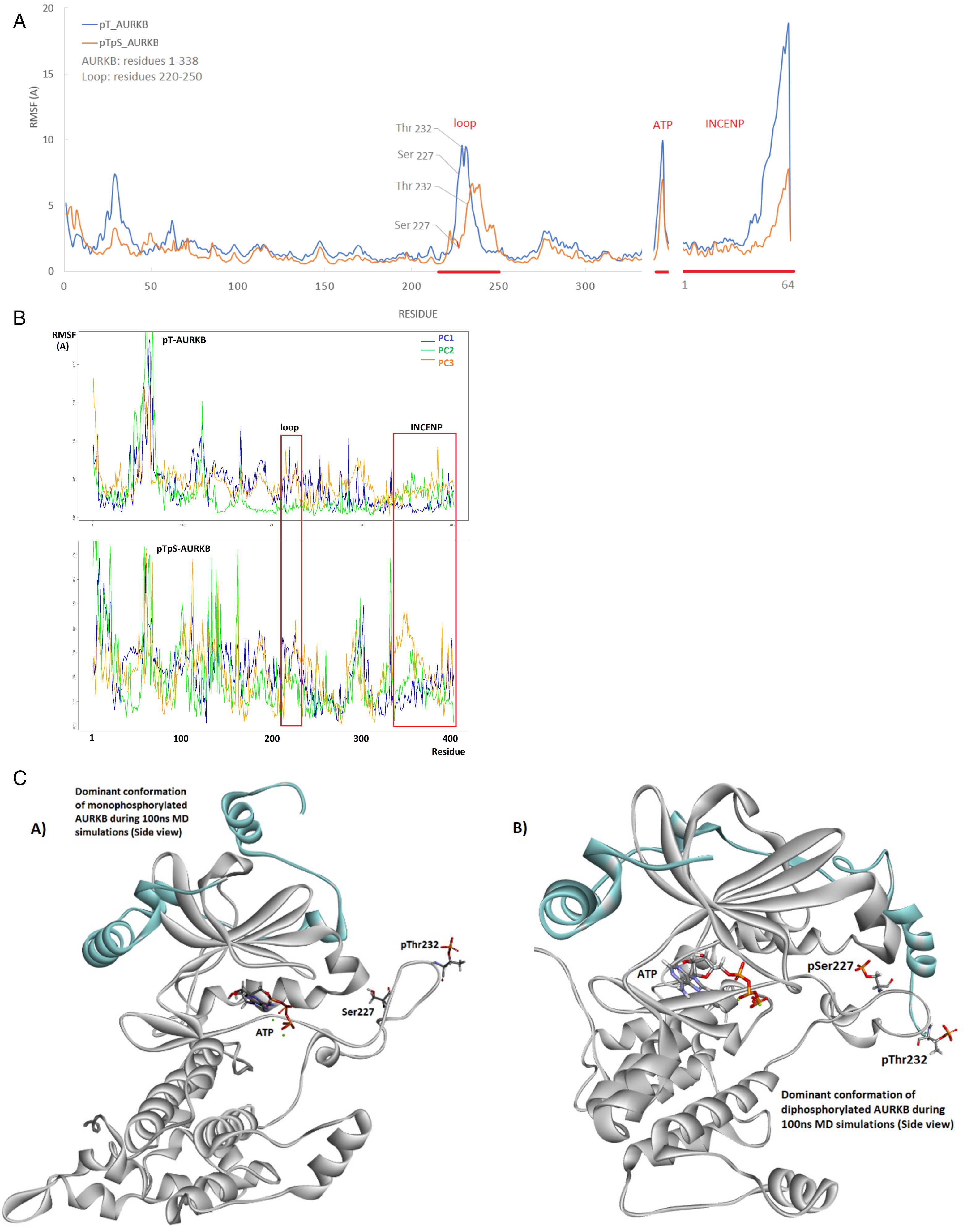

Supplementary Figure 6.
(A) RMSF plot showing the dynamics and flexibility of different regions of pT232 and pS227/pT232 Aurora B kinase during the course of 100ns MD simulation. (B) Principal component (PC) analysis of 100ns MD simulation of Aurora B pT232 (upper panel) and Aurora B pS227/pT232 (lower panel). (C) Dominant conformations of Aurora B pT232 (left panel) and pS227/pT232 (right panel) extracted from the trajectories of 100ns MD simulation.

Supplementary Figure 7

A

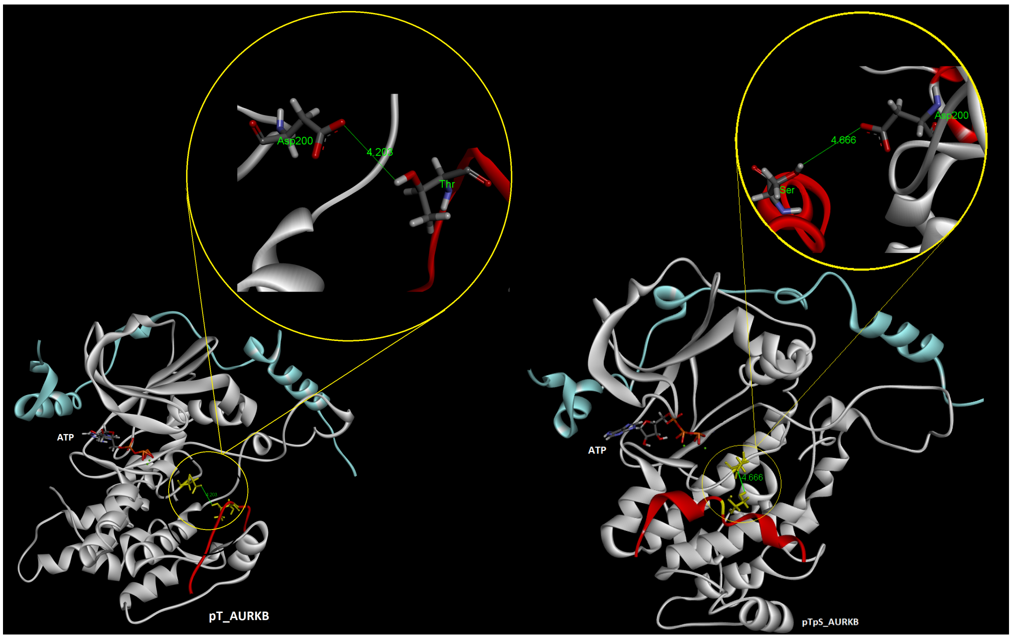

B

Distance (A)	TopoIIα S29 Peptide: QKPDPAKTKNRRKRKP		TopoIIα T1460 Peptide: NEDAKKRLSVERIYQKK	
	Before MD	After MD	Before MD	After MD
pT232 AKB-0	10.768	4.203	12.539	8.305
pS227/pT232 AKB-1101	18.764	12.598	12.948	4.666

C

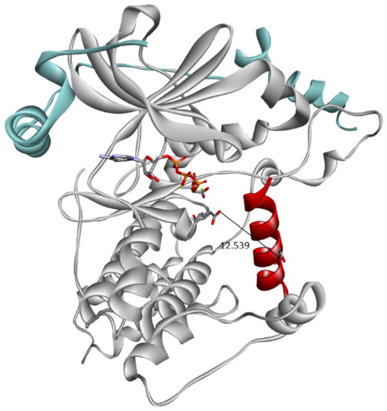

pT232 AurKB with TopoIIα S29 peptide
Before MD Simulation

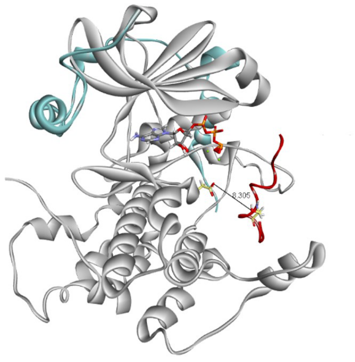

pT232 AurKB with TopoIIα S29 petide
After MD Simulation

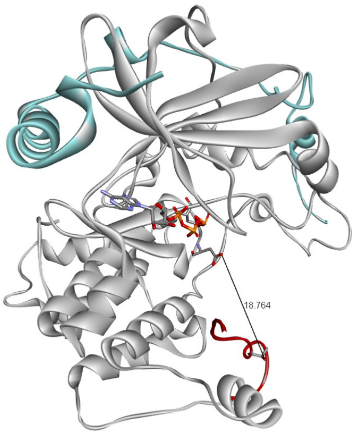

pS227/pT232 AurKB with T1460 peptide
Before MD Simulation

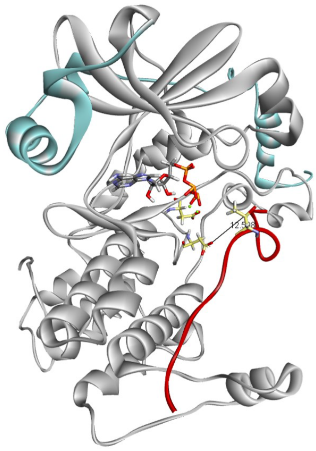

pS227/pT232 AurKB with T1460
After MD Simulation

Supplementary Figure 7.
(A) Docked poses of substrate peptides. Aurora B pT232 with TopoIIα pT1460 peptide (left panel) and Aurora B pS227/pT232 with TopoIIα pS29 peptide (right panel). (B) Table of distances between D200 of HRD motif and OH group of target amino acid (Thr and Ser residues). (C) Docked poses of Aurora B phospho-isoforms with non-substrate peptides before and after MD simulation.

Supplementary Figure 8

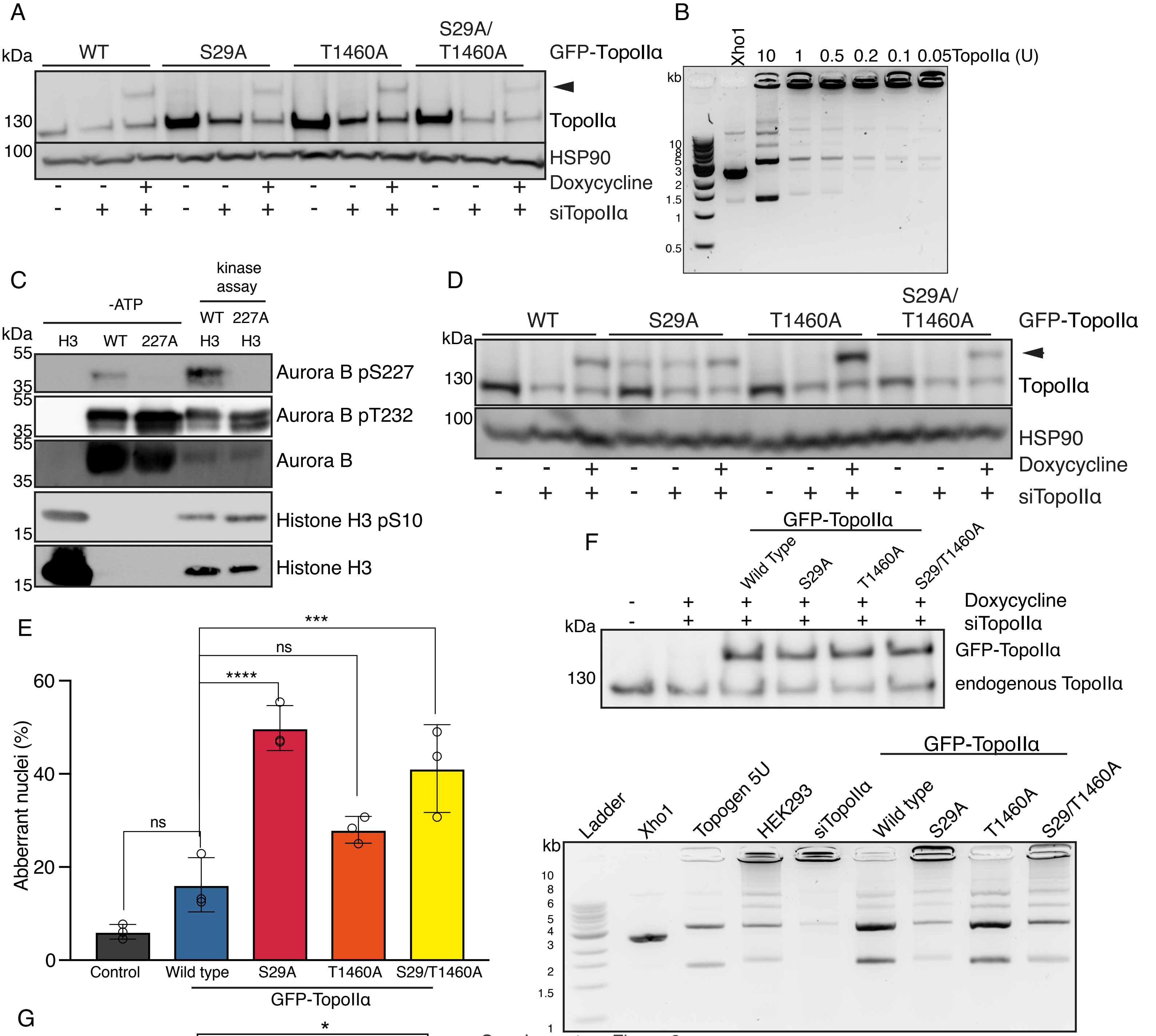

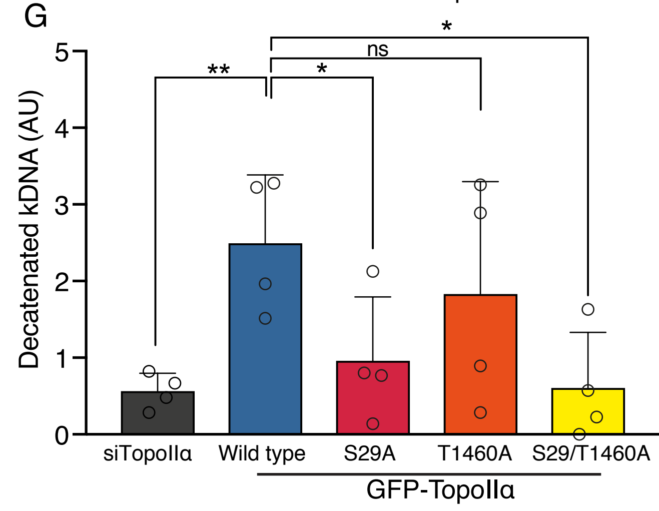

Supplementary Figure 8.
(A) DLD1-FlpIn-Trex cell lines were developed to express doxycycline inducible GFP-TopoIIα wild type, S29A, T1460A and S29/T1460A. Endogenous TopoIIα was depleted using a siRNA specific to the 3' UTR. Immunoblot is representative of 3 experiments. (B) DNA gel (representative of 3 experiments) showing decreasing amounts of TopoIIα to determine sub-optimal threshold and decatenated kDNA. (C) In vitro kinase assay of recombinant Aurora B WT and S227A against known Aurora B substrate Histone H3 S10 demonstrating activity of both phospho-species of the kinase. Immunoblot is representative of 3 experiments. (D) HEK293-FlpIn-Trex cell lines were developed to express doxycycline inducible GFP-TopoIIα wild type, S29A, T1460A and S29/T1460A. Endogenous TopoIIα was depleted using a siRNA specific to the 3' UTR. Immunoblot is representative of 3 experiments. (E) HEK293 cells induced to express GFP-TopoIIα wild type or mutants were scored for aberrant nuclear morphology. The mean ±SEM of three independent experiments is represented by the graph (n=>200) (one-way ANOVA, ***P=0.0008****P=≤0.0001; Control 6.1%±0.9, WT 16.2%±3.36, S29A 49.83%±2.79, T1460A29%±1.65, S29/T1460A 41.17%±5.44). (F) TopoIIα was extracted from HEK293 cells were induced to express GFP-TopoIIα. Amounts of TopoIIα were equalised by immunoblot (upper panel) before in vitro decatenation assay (lower panel) (n=4) . The amount of decatenated kDNA present after addition of extracted TopoIIα was quantified by ImageJ. (G) Graph represents the mean ±SD of four independent experiments. (one-way ANOVA; *P=0.011 (S29/T1460A), *P=0.032 (S29A), **P=0.0095(siTopoIIa) siTopoIIa 0.57±0.23, WT 2.49±0.89, S29A 0.96±0.83, T1460A 1.83±1.46, S29/T1460A 0.61±0.72).

Supplementary Figure 9 - uncropped blots

Figure 1F

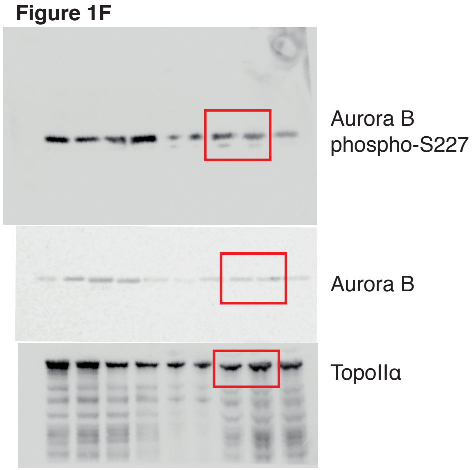

Figure 2B

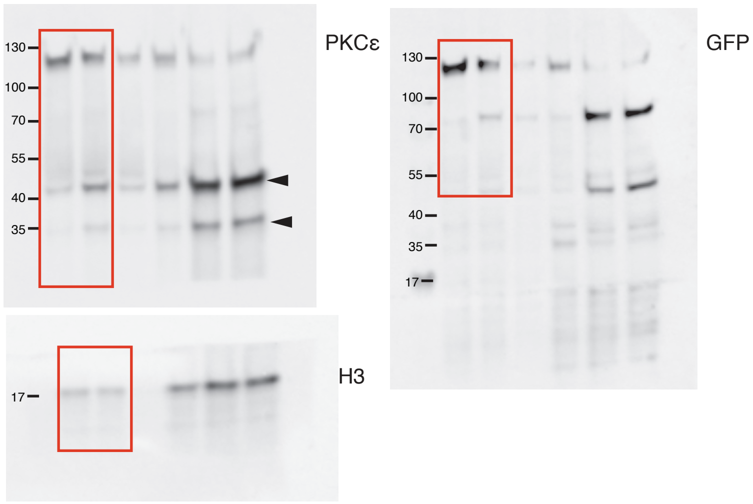

Figure 2D

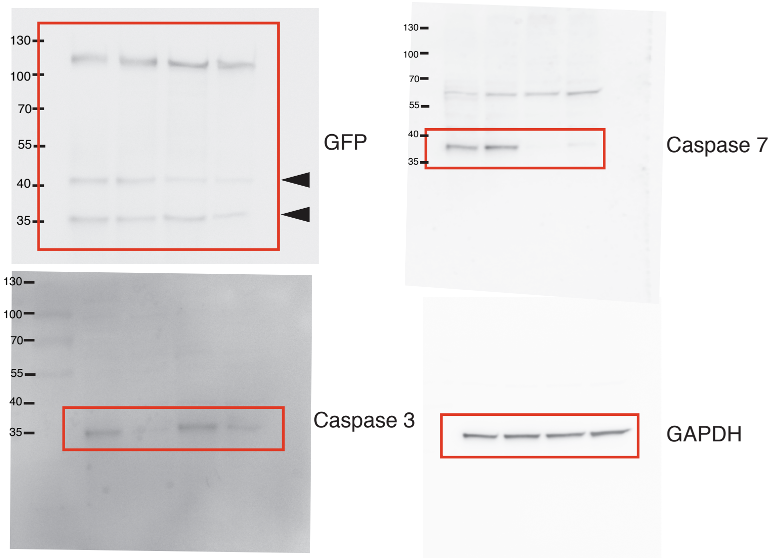

Figure 2E

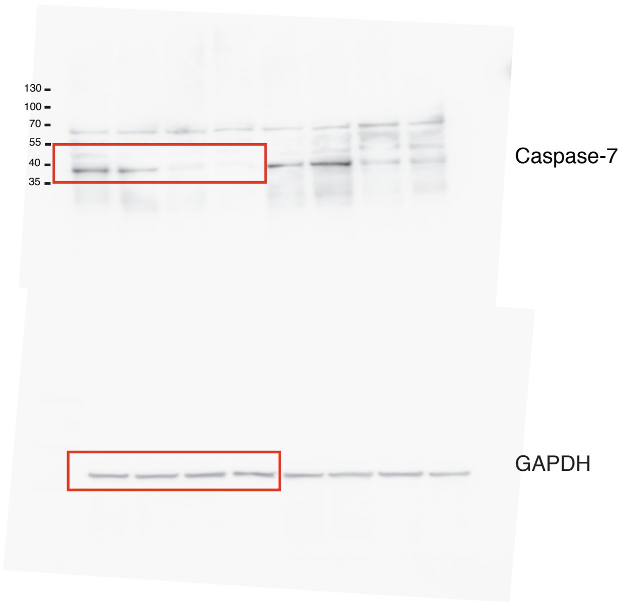

Figure 2F

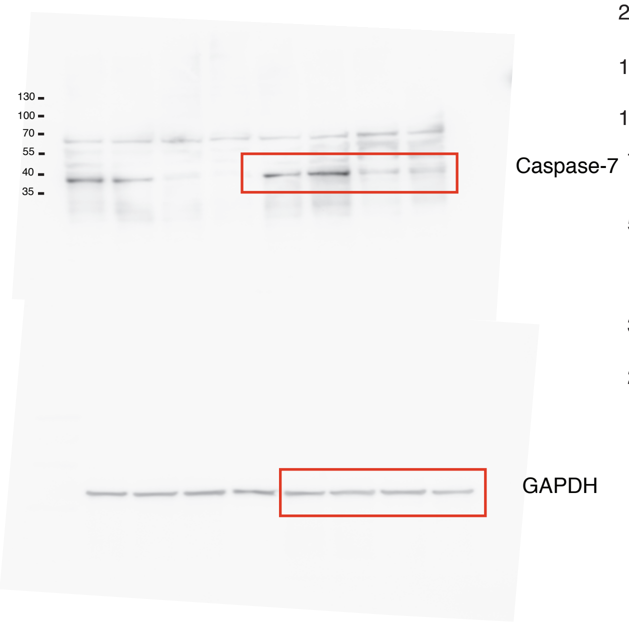

Figure 3H

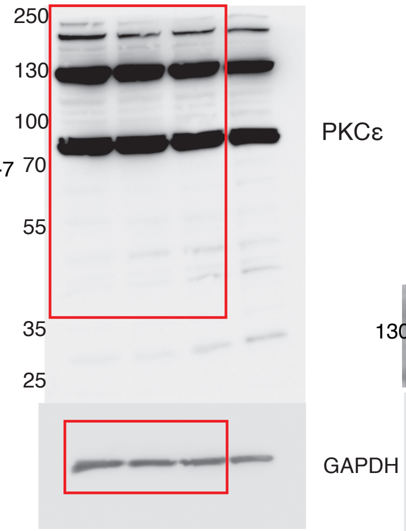

Figure 4E

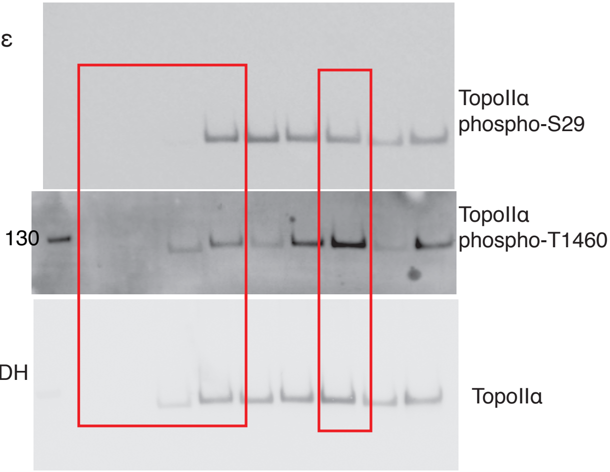

Supplementary Figure 1A

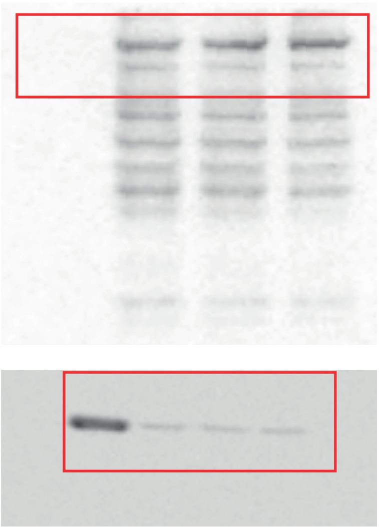

Supplementary Figure 1B

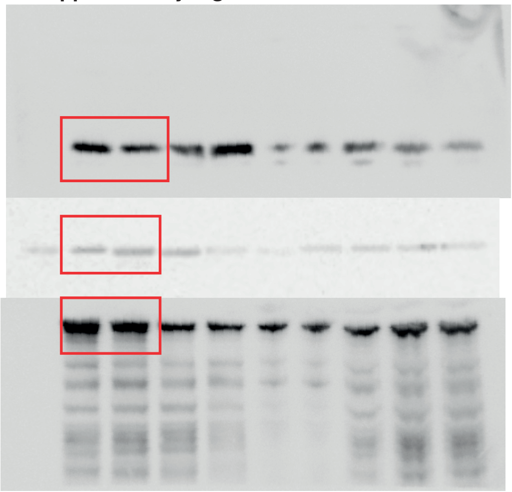

Supplementary Figure 1C

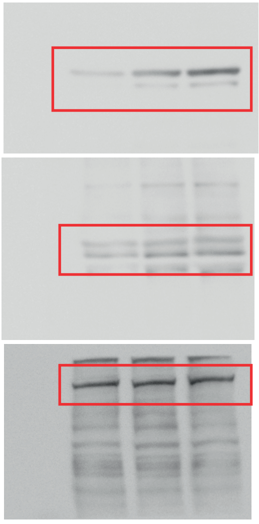

Supplementary Figure 2A

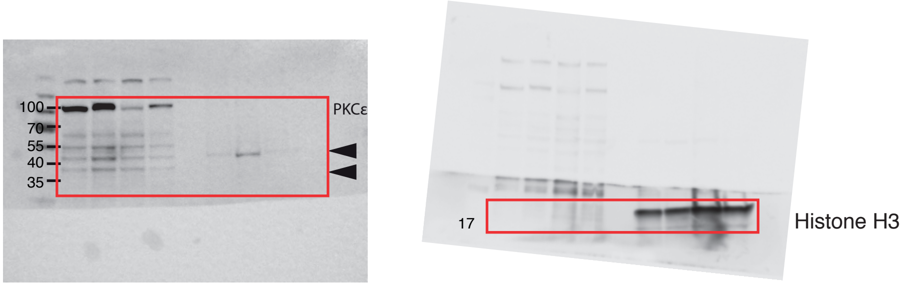

Supplementary Figure 2B

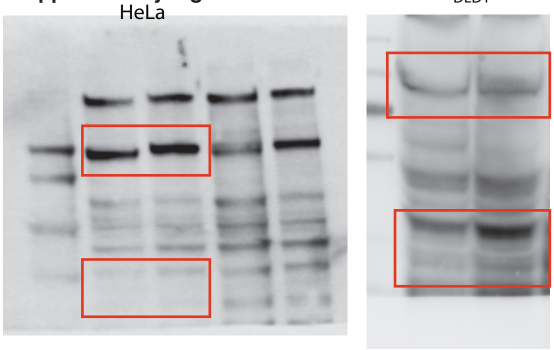

Supplementary Figure 2C

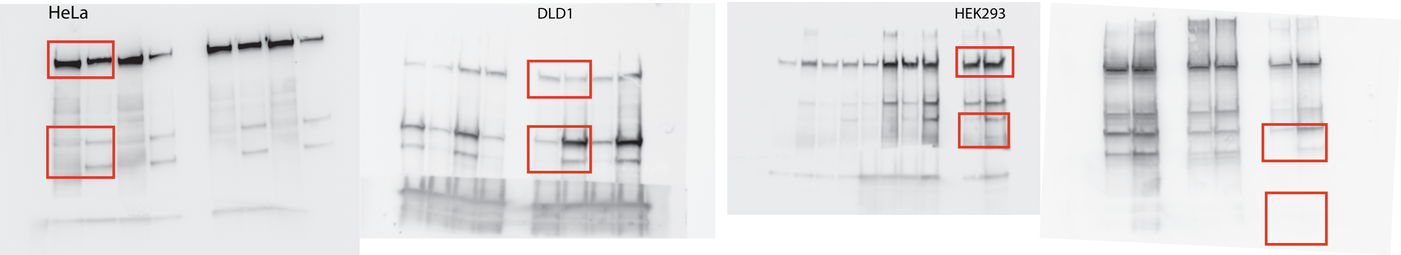

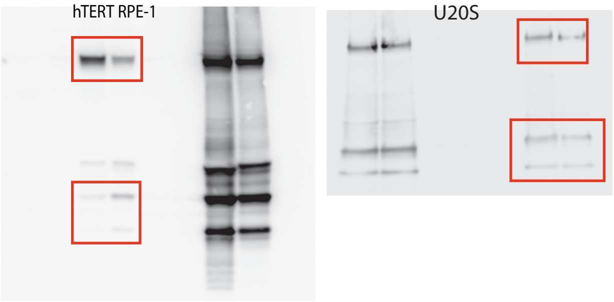

Supplementary Figure 2G

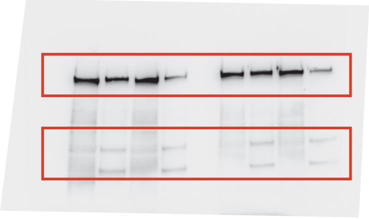

Supplementary Figure 3A

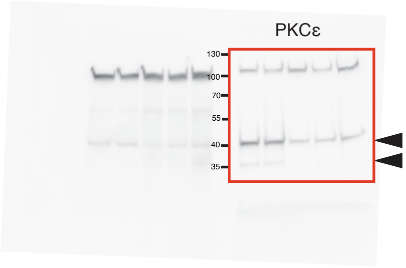

Supplementary Figure 2H

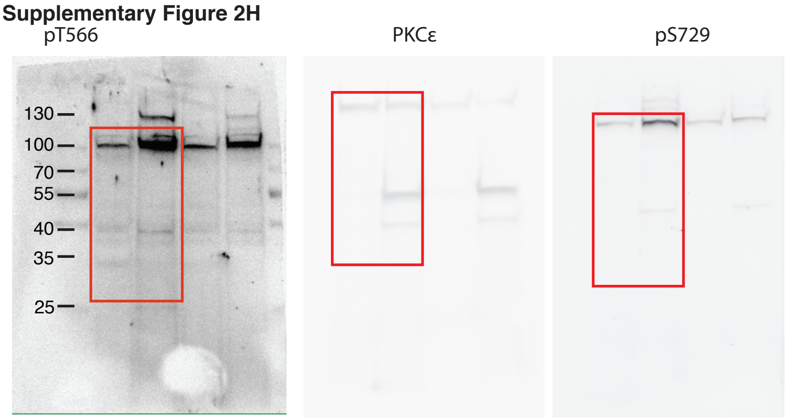

Supplementary Figure 3C

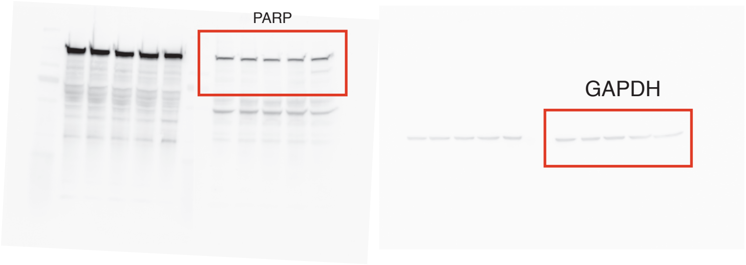

Supplementary Figure 3D

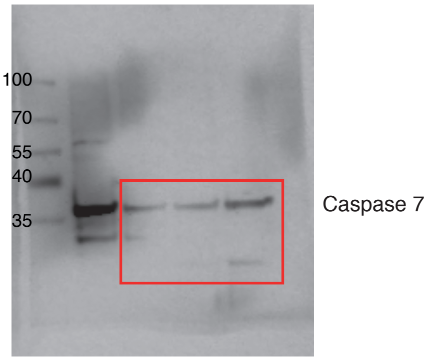

Supplementary Figure 4A

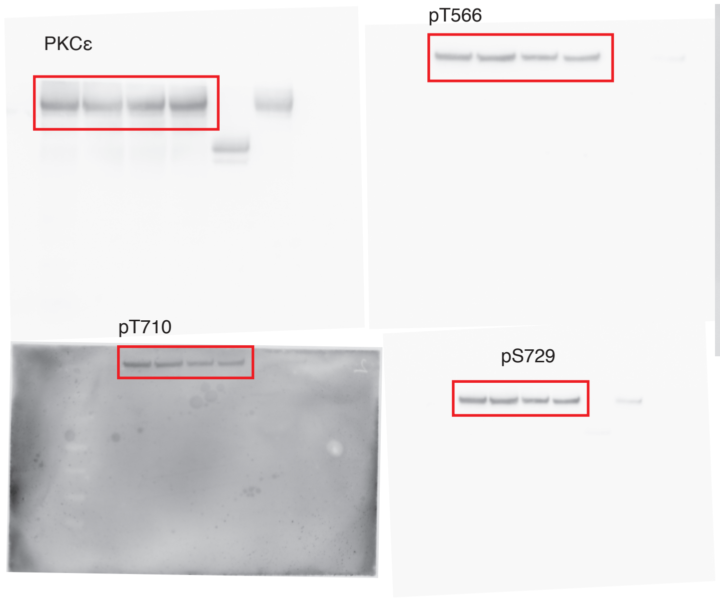

Supplementary Figure 4C

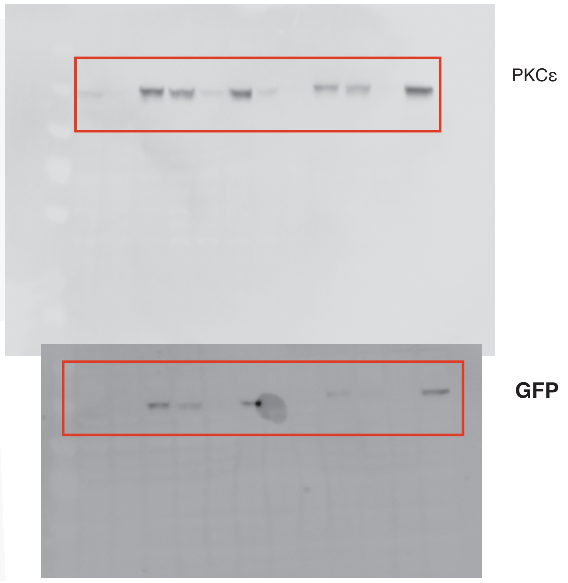

Supplementary Figure 5C

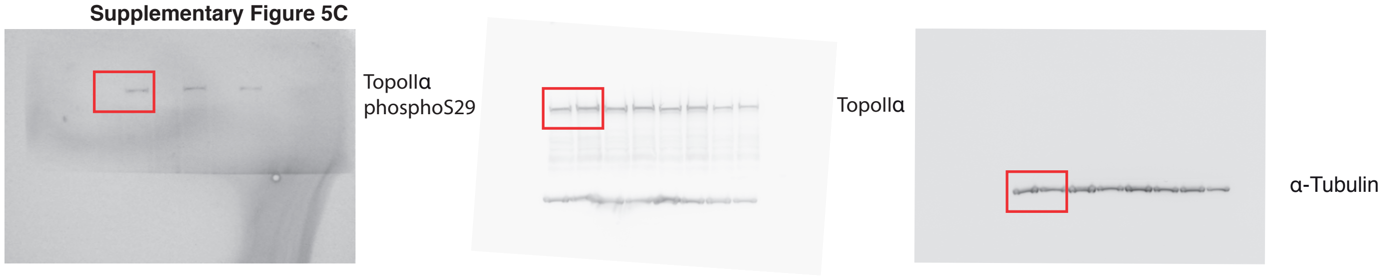

Supplementary Figure 5E

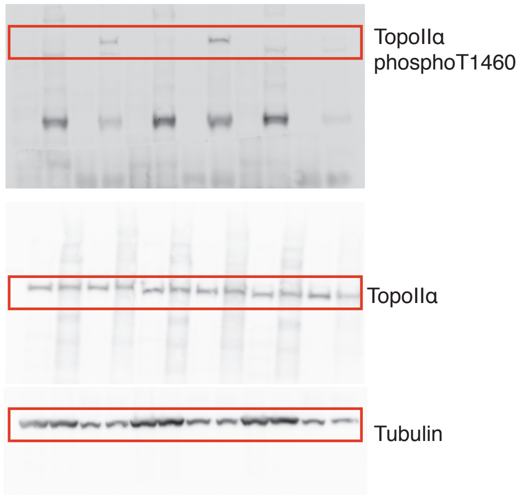

Supplementary Figure 8A

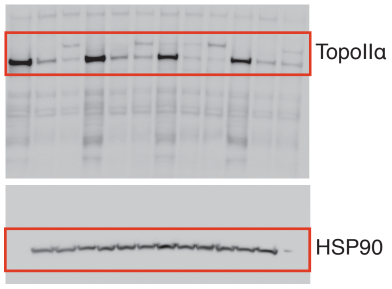

Supplementary Figure 8C

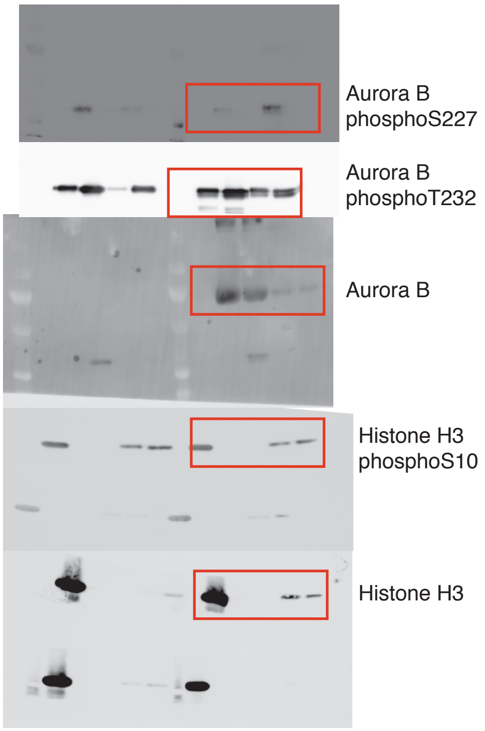

Supplementary Figure 8D

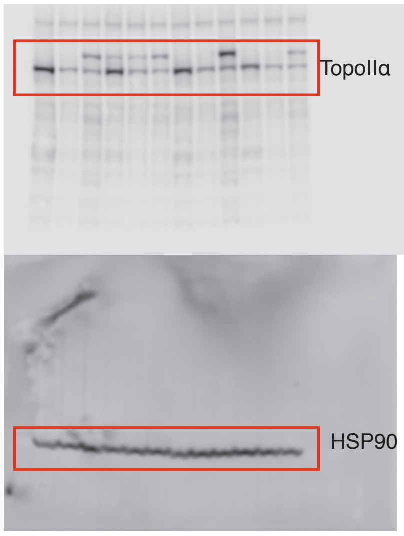

Supplementary Figure 8F

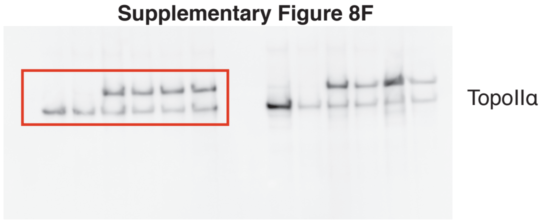

Supplement: Supplementary file 1 — Supplementary Information [file 41467_2020_15163_MOESM1_ESM.pdf]
